# Synthesis, computational studies, tyrosinase inhibitory kinetics and antimelanogenic activity of hydroxy substituted 2-[(4-acetylphenyl)amino]-2-oxoethyl derivatives

**DOI:** 10.1080/14756366.2019.1654468

**Published:** 2019-08-28

**Authors:** Muhammad Rafiq, Yasir Nazir, Zaman Ashraf, Hummera Rafique, Samina Afzal, Amara Mumtaz, Mubashir Hassan, Anser Ali, Khurram Afzal, Muhammad Rizwan Yousuf, Muhammad Saleem, Katarzyna Kotwica-Mojzych, Mariusz Mojzych

**Affiliations:** a Department of Physiology & Biochemistry, Cholistan University of Veterinary and Animal Sciences, Bahawalpur, Punjab, Pakistan;; b Department of Chemistry, Allama Iqbal Open University, Islamabad, Pakistan;; c Department of Chemistry, University of Gujrat, Gujrat, Pakistan;; d Faculty of Pharmacy, Bahauddin Zakria University, Multan, Pakistan;; e Department of Chemistry, COMSAT University Islamabad, Abbottabad, Pakistan;; f Department of Biology, College of Natural Sciences, Kongju National University, Gongju, Korea;; g Department of Zoology, Mirpur University of Science and Technology (MUST), Mirpur, Pakistan;; h Department of Theriogenology, University of Veterinary and Animal Sciences, Lahore, Pakistan;; i Department of Chemistry, University of Sargodha, Bhakkar, Pakistan;; j Department of Histology and Embryology with Experimental Cytology Unit, Medical University of Lublin, Lublin, Poland;; k Department of Chemistry, Siedlce University of Natural Sciences and Humanities, Siedlce, Poland

**Keywords:** Tyrosinase inhibition, enzyme inhibitory kinetics, molecular docking, antimelanogenic activity, cytotoxicity

## Abstract

The over expression of melanogenic enzymes like tyrosinase caused many hyperpigmentaion disorders. The present work describes the synthesis of hydroxy substituted 2-[(4-acetylphenyl)amino]-2-oxoethyl derivatives **3a–e** and **5a–e** as antimelanogenic agents. The tyrosinase inhibitory activity of synthesized derivatives **3a–e** and **5a–e** was determined and it was found that derivative **5c** possesses excellent activity with IC_50_ = 0.0089 µM compared to standard kojic acid (IC_50_ = 16.69 µM). The presence of hydroxyl groups at the *ortho* and the *para* position of cinnamic acid phenyl ring in compound **5c** plays a vital role in tyrosinase inhibitory activity. The compound **5d** also exhibited good activity (IC_50_ = 8.26 µM) compared to standard kojic acid. The enzyme inhibitory kinetics results showed that compound **5c** is a competitive inhibitor while **5d** is a mixed-type inhibitor. The mode of binding for compounds **5c** and **5d** with tyrosinase enzyme was also assessed and it was found that both derivatives irreversibly bind with target enzyme. The molecular docking and molecular dynamic simulation studies were also performed to find the position of attachment of synthesized compounds at tyrosinase enzyme (PDB ID 2Y9X). The results showed that all of the synthesized compounds bind well with the active binding sites and most potent derivative **5c** formed stable complex with target protein. The cytotoxicity results showed that compound **5c** is safe at a dose of 12 µg/mL against murine melanoma (B16F10) cells. The same dose of **5c** was selected to determine antimelanogenic activity; the results showed that it produced antimelenogenic effects in murine melanoma (B16F10) cells. Based on our investigations, it was proposed that compound **5c** may serve as a lead structure to design more potent antimelanogenic agents.

## GRAPHICAL ABSTRACT

**Figure:**
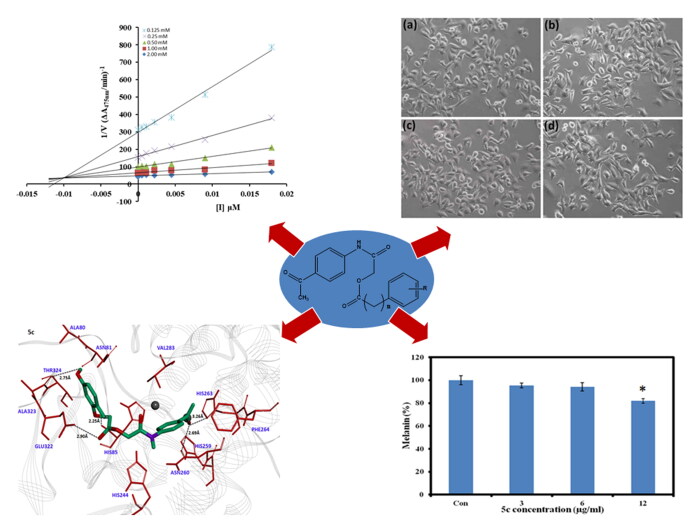

## 1. Introduction

Melanogenesis is the process of melanin synthesis; most often produced by cells called melanocytes^1^^,^^2^. Melanocytes are dendritic cells formed from melanoblasts which are unpigmented cells originating from embryonic neural crest cells^3^^,^^4^. Melanocytes are found in the basal layer of skin epidermis and hair follicles. The primary function of melanocytes is the synthesis of melanin pigment. In skin each melanocytes is surrounded by approximately 36 keratinocytes^5^^,^^6^, to which they transfer their synthesized melanin^6^^,^^7^.

Melanin is a pigment, primarily responsible for skin colour. It retains antioxidative and photoscreening effect, therefore, provides skin photo-protection, prevents from injury and, absorbs and transforms harmful UV radiations into harmless heat^8^. Despite its advantages, increased production and accumulation of pigmentations can cause skin problems such as freckles, age spots, post-inflammatory hyperpigmentation, lentigo and melanoma. UV which stimulates melanin synthesis is reported to cause gene mutation, DNA damage, impaired immune system and cancer^9^. Pigmentation in metastatic melanoma patients results in short overall and disease-free survival^10^. Melanin content is correlated with higher disease stage and seems to protect malignant melanocytes from chemo-, radio- and photodynamic therapy^11^^,^^12^. Therefore, inhibition of melanogenesis could be a rational approach for controlling metastatic melanoma, abnormal skin pigmentation and related disorders^10^.

Tyrosinase is key an enzyme showing the rate limiting affect in melanin biosynthesis. In cytosol, L-phenylalanine may be converted to tyrosine by phenylalanine hydroxylase (PAH) in order to serve as the substrate for tyrosinase^13^. Tyrosinase also catalyses the hydroxylation of L-tyrosine to 3,4-dihydroxyphenylalanine (L-DOPA) and L-DOPA to dopaquinones^14^ which under unregulated conditions result in abnormal accumulation of melanin pigments^15^^,^^16^. Therefore, inhibition of tyrosinase is the simplest approach and tyrosinase inhibitors may be an attractive target to achieve depigmentation.

Additionally, antioxidants are reported critical for melanin inhibition. Reactive oxygen species (ROS) and free radicals are shown to contribute in numerous skin disorders including hyperpigmentation^17^. During hyperpigmentation, increased level of hydrogen peroxide (H_2_O_2_) and other ROS is reported which may increase the load of oxidative stress on melanocytes. Numerous ROS scavengers and inhibitors of ROS generation are shown to interfere with oxidation processes, therefore, frequently used to reduce oxidative damage in human body and to downregulate UV-induced melanogenesis^17–20^.

**Figure 1.**
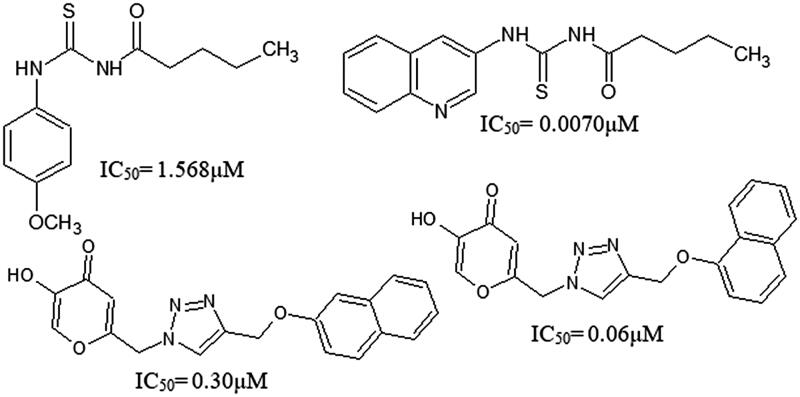
Structures of recently reported tyrosinase inhibitors.

For melanin inhibition, potent tyrosinase inhibitors and antioxidant agents are always desirable. They can be obtained from a variety of sources; however, safety concerns pose a big challenge for their commercialization. There are a number of tyrosinase inhibitors; hydroquinone and kojic acid and, potent antioxidants; *tert*-butyl hydroxyanisole (BHA) and *tert*-butyl hydroxytoluene (BHT) which may show undesirable side effects; cytotoxicity, dermatitis and skin cancer^21^. Recently, methoxy phenyl thiourea, thiazole derivatives and kojic acid derivatives have been reported as the tyrosinase inhibitor (Figure 1)^22–24^. Therefore, search for safe and effective depigmenting agents is required which may address these issues and serve as a better solution for treating pigment related dermatological disorders.

Keeping in view the importance of phenolic hydroxyl in tyrosinase inhibition,^25^^,^^26^ the present work is planned to synthesize hydroxy substituted 2-[(4-acetylphenyl)amino]-2-oxoethyl derivatives **3a–e** and **5a–e** to explore the role of different functionalities in tyrosinase inhibitory activity. The enzyme inhibitory kinetics of most potent compounds was determined by Line-weaver Burk Plots and Dixon Plots. The reversibility of enzyme–inhibitor complex was also determined. The computational molecular docking of the synthesized compounds was performed against target protein PDBID 2Y9X to predict the binding sites of these compounds in target protein. The molecular dynamic simulation was also performed to check the stability of enzyme-inhibitor complex computationally. The cytotoxicity and antimelanogenic activity of most potent derivative **5c** was also performed against murine melanoma (B16F10) cells.

## 2. Results and discussion

### 2.1. Chemistry

**Scheme 1.**
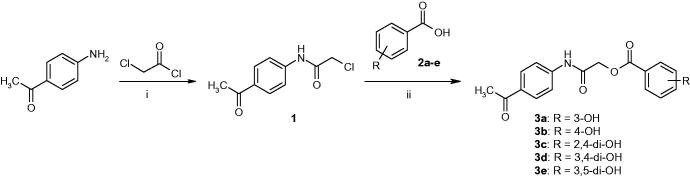
Synthesis of compounds **3a–e.** Reagents and conditions: (i) CH_2_Cl_2_/Et_3_N, 0–5 °C, reflux for 5 h; (ii) DMF/Et_3_N/KI, r.t., reflux for 24 h.

**Scheme 2.**
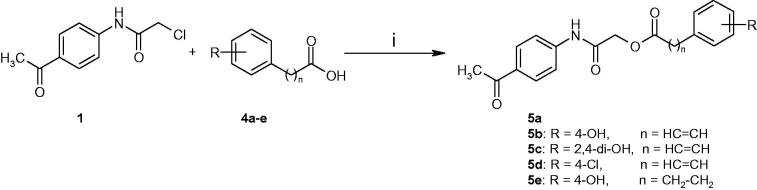
Synthesis of compounds **5a–e**. Reagents and conditions: (i) DMF/Et_3_N/KI, r.t., reflux for 24 h.

The title compounds **3a–e** and **5a–e** were synthesized by the following already reported method^27^^,^^28^ as shown in Schemes 1 and 2. 4-Acetyl aniline was reacted with chloroacetyl chloride in the presence of triethyl amine to synthesized intermediate **1**. The final products **3a–e** and **5a–e** were synthesized by reacting substituted benzoic acids and cinnamic acids with intermediate **1**. The formation of the final products was ascertained by their FTIR, ^1^HNMR and ^13^CNMR spectral data. The absorption for amide –NH stretching appeared at 3133–3156 cm^−1^ in FTIR spectra while amide carbonyl appeared at 1632–1656 cm^−1^. The amide –NH in ^1^HNMR spectra appeared as a singlet at downfield region, rest of the signals are present in the acceptable regions.

### 2.2. Enzyme inhibitory kinetics

Hydroxy substituted 2-[(4-acetylphenyl)amino]-2-oxoethyl derivatives **3a–e** and **5a–e** have been designed and synthesized to explore their inhibitory effects on tyrosinase activity. Kojic acid a clinically applied agent was used as a standard for comparison purpose. The presence of phenolic hydroxyls is of special interest because of their high tyrosinase inhibitory activity. It has been exposed from our bioassay results that the major determining factor of inhibitory activity is the presence of phenolic hydroxyls at cinnamic acid moiety (Table 1). Interestingly, compound **5c** bearing 2,4-dihydroxy substituted cinnamic acid moiety exhibited excellent activity (IC_50_ = 0.0089 µM) and is also more active than standard kojic acid (IC_50_ = 16.69 µM). The compound **5d** also exhibited good activity (IC_50_ = 8.26 µM) compared to standard kojic acid. The bioassay results proved that derivatives with hydroxy substituted cinnamic acid moiety (**5a–d**) displayed greater tyrosinase inhibitory activity compared to hydroxy substituted benzoic acid derivatives (**3a–e**).

**Table 1.** Tyrosinase inhibitory activity of the synthesized compounds **3a–e** and **5a–e**.

Compounds	Mushroom tyrosinase inhibition IC_50_ (µM)
**1**	54.5 ± 3.1
**3a**	324.6 ± 16.9
**3b**	319.5 ± 31.9
**3c**	145.2 ± 16.7
**3d**	256.8 ± 23.4
**3e**	165.9 ± 14.2
**5a**	63.5 ± 2.5
**5b**	97.3 ± 5.8
**5c**	0.0089 ± 0.0004
**5d**	8.26 ± 1.7
**Kojic acid**	16.69 ± 2.8

To understand the mechanism of mushroom tyrosinase inhibition by compounds **5c** and **5d,** kinetic studies were conducted. Kinetic studies showed a concentration-dependent mushroom tyrosinase inhibition by tested inhibitors. Constant and continual monitoring of the reaction showed a marked decrease of the reaction rate in the presence of the inhibitors, which ultimately indicated the decrease of the final absorbance when compared with controls without inhibitor. The potency of inhibition exhibited by these compounds changed depending on the presence and position of substituents and on the class of compounds^29^. Enzyme inhibition kinetics was analysed by Lineweaver**–**Burk plot and Dixon plots to determine the type of inhibition and inhibition constant (*K_I_*).

**Figure 2.**
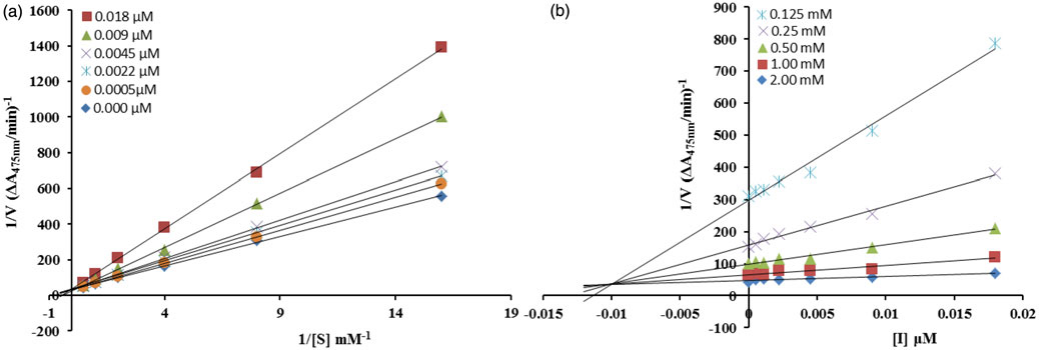
**(**a**)** Lineweaver–Burk plots for the inhibition of the diphenolase activity of mushroom tyrosinase by various concentrations 0.000, 0.0005, 0.0022, 0.0045, 0.009 and 0.018 µM of compound **5c** in the presence of different concentrations 0.062, 0.125, 0.25, 0.5, 1 and 2 mM, of L-DOPA. (b) Dixon plots for the inhibition of the diphenolase activity of mushroom tyrosinase by various concentrations of compound **5c** in the presence of different concentrations 0.125, 0.25, 0.5, 1 and 2 mM, of L-DOPA.

**Figure 3.**
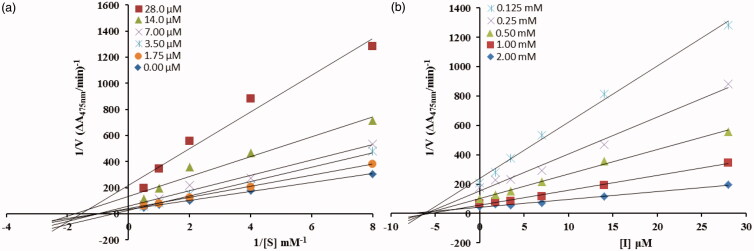
(a) Lineweaver–Burk plots for the inhibition of the diphenolase activity of mushroom tyrosinase by various concentrations 0.00, 1.75, 3.5, 7, 14 and 28 µM of compound **5d** in the presence of different concentrations 0.125, 0.25, 0.5, 1 and 2 mM, of L-DOPA. (b**)** Dixon plots for the inhibition of the diphenolase activity of mushroom tyrosinase by various concentrations of compound **5d** in the presence of different concentrations 0.125, 0.25, 0.5, 1 and 2 mM, of L-DOPA.

From the kinetic analyses, the Lineweaver–Burk plot of *1/V*_max_ versus 1/[S] in the presence of different concentrations of **5c** showed that *K_m_* value change while that of *1/V*_max_ remained the same and in case of compound **5d** both the *K_m_* and *1/V*_max_ reached to new values as shown in Figures 2(a) and 3(a), respectively. This graphical representation showed that the compound **5c** is a competitive inhibitor and the compound **5d** is a mixed-type inhibitor. On the other hand, in the Dixon plot, the dissociation constant *Ki* value for **5c** with 0.01 µM and for **5d** with 6 µM was calculated from the plots for uninhibited enzyme and with different concentrations of inhibitors as shown in Figures 2(b) and 3(b), respectively.

**Figure 4.**
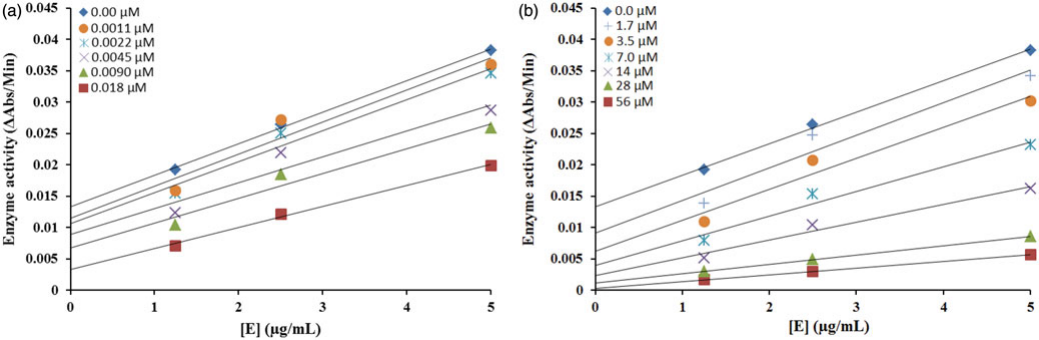
Relationship between the catalytic activity of tyrosinase and various concentrations of compound **5c** shown in (a) and **5d** shown in (b).

The inhibitory mechanism of mushroom tyrosinase by compounds **5c (**in concentrations: 0.0, 0.0011, 0.0022, 0.0045, 0.0090 and 0.018 µM) and **5d (**in concentrations: 0.0, 1.75, 3.5, 7.0, 14, 28 and 56 µM) were investigated. The plots of the remaining enzyme activity versus the concentration of enzyme (1.25, 2.5 and 5 µg/mL) at different inhibitor concentrations for the catalysis of L-DOPA gave a series of parallel straight lines with the same slopes indicating that the inhibitory effect of **5c** on the tyrosinase was irreversible (Figure 4(a)). Identical research was carried out for compound **5d** and the same conclusion was drawn that **5d** was irreversible inhibitor (Figure 4(b)). These results suggested that both the compounds effectively inhibited the enzyme by binding to its binuclear active site irreversibly^30^.

### 2.3. Cytotoxicity and antimelanogenic activity

**Figure 5.**
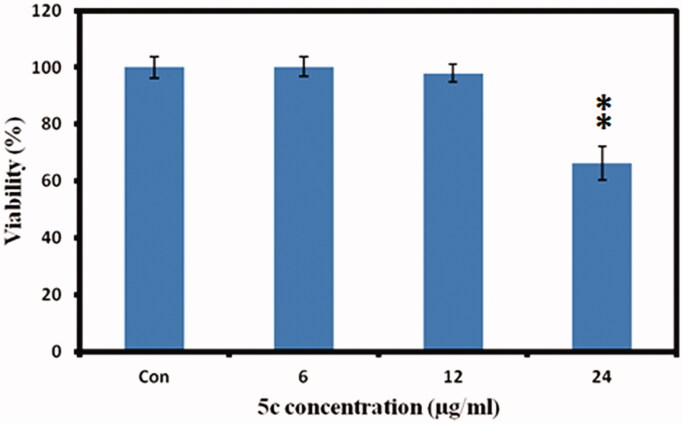
Cell viability measurements. The murine melanoma (B16F10) cells were incubated with indicated concentrations of **5c**, and the cell viability was measured by MTT assay. The data was expressed as a percentage of the control (normalized to 100%) with mean ± standard deviation and was analysed using Student’s *t*-tests, **p < 0.005.

**Figure 6.**
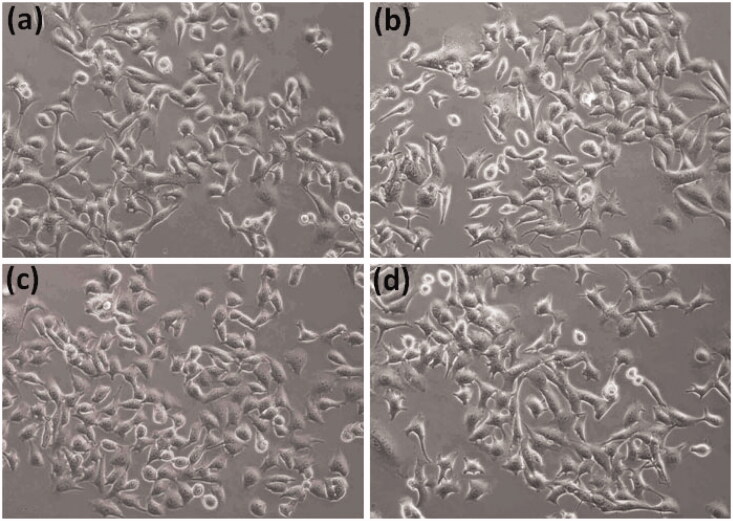
The B16F10 cells morphology. The cells were incubated with (a) cell culture medium, (b) 3 µg/ml, (c) 6 µg/ml, and (d) 12 µg/ml concentrations of **5c** for 24 h and then cell images were taken by inverted fluorescent microscope (Nikon, ECLIPSE, Ti, Tokyo, Japan).

To investigate the effect of compound **5c** on cellular melanin synthesis, we first assessed the safe treatment dose of tested derivative **5c** using MTT cell viability assay. For viability assay, murine melanoma (B16F10) cells were incubated with various concentrations of compound **5c** for 24 h and then MTT assay was performed as described in the methods section. The result showed concentration dependent **5c** effect on cell viability as shown in Figure 5. The **5c** compound showed significant toxic effect (33.7% compared to control normalized to 100%) at 24 µg/ml concentrations however no significant effect at 12 µg/ml concentration was observed. Therefore, we selected 12 µg/ml concentration (safe concentration) of **5c** for further analysis. Later, we took cellular images which showed no effect on cell morphology after incubation with **5c** (Figure 6).

**Figure 7.**
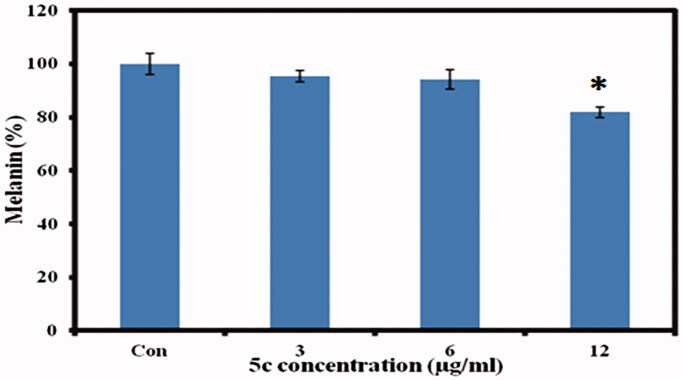
Intracellular melanin measurements. The murine melanoma (B16F10) cells were incubated with indicated concentrations of **5c**, and the cellular melanin content was detected. The data were expressed as a percentage of control (normalized to 100%) with mean ± standard deviation and, and were analysed using Student’s *t*-tests, *p < 0.05.

For melanin measurement, B16F10 cells were incubated in cell culture medium containing various concentrations of **5c** for 24 h. Interestingly, cellular melanin analysis showed decrease of melanin content with increasing concentration of **5c** (Figure 4). The significant decrease of cellular melanin content was observed at 12 µg/ml where we got 82% melanin content as compared to control normalized to 100% as given in Figure 7. The melanoma cells are widely used for melanin investigations and various studies have shown the effect of compound concentration on melanogenesis^31–35^. In conclusion, our results indicate pronounced ability of **5c** compound to inhibit melanin synthesis in cells *in vitro* which may show promising future in the field of cosmetics or medicine.

### 2.4. Structural assessment of target protein

Mushroom tyrosinase (Agaricusbisporus) is a class of oxidoreductase copper containing protein comprises 391 amino acids. The structure architecture of mushroom tyrosinase showed that it consists of 39% helices (154 residues) and 14% β sheets (57 residues) and 46% coils (180 residues). The X-ray diffraction study confirmed its resolution 2.78Ȧ, *R*-value 0.238 and unit cell crystal dimensions like length and angles of coordinates. The unit cell length values were observed for *a* = 103.84, *b* = 104.82 and *c* = 119.36 with angles 90°, 110.45° and 90° for all α, β and γ dimensions, respectively. The Ramachandran plots and values indicated that 95.90% of residues were in favoured regions and 100.0% residues were lies in allowed regions (Figure S1). This selected Ramachandran graph values showed the good accuracy of phi (φ) and psi (ψ) angles among the coordinates of receptor molecules and most of residues plunged in acceptable region.

### 2.5. Chemo-informatics properties and Lipinski’s rule

The predicted chemo-informatics properties such as molar volume, density, polarizability and surface tension were evaluated by computational approach. Literature study established a standard value for molar refractivity (40–130), molecular weight (160–480) and number of atoms (20–70) ^36^. Results showed that **5c** predicted values are much better than standard values and all other synthesized compounds. Moreover, the Lipinski’s rule of five (RO5) says nothing about specific chemistry or structural features found in drugs or non-drugs. The computational results showed that **5c** possesses 6 HBA, 3 HBD and 2.54 LogP values which significantly justified its drug like behaviour. Moreover, their molecular weight (355.11 g/mol) was also much better than standard value (<5000 g/mol). The RO5 justifies that molecules with poor absorption are more likely to have more than 5 HBD, MWT over 500, logP over 5 and more than 10 HBA. However there are plenty of examples available for RO5 violation amongst the existing drugs^37^. In overall results, these predicted values justify the significance of **5c** synthesized compound as a good candidate molecule (Table 2).

**Table 2.** Chemo-informatics evaluation of synthesized compounds.

Properties	**3a**	**3b**	**3c**	**3d**	**3e**	**5a**	**5b**	**5c**	**5d**	**5e**
MW	313	313	329	329	329	323	339	355	357	341
HBA	5	5	6	6	6	4	5	6	4	5
HBD	2	2	3	3	3	1	2	3	1	2
LogP	2.16	2.16	1.78	1.78	1.90	3.18	2.92	2.54	3.89	2.43
LogS	61.44	64.12	124.89	85.62	92.75	6.53	9.53	23.64	0.80	75.09
PSA	75.69	75.69	92.24	91.17	93.31	57.48	75.09	91.64	57.48	75.09
MR	83.77	83.77	85.66	85.66	85.66	92.42	94.30	96.18	97.31	92.59
Den.	1.33	1.33	1.41	1.41	1.41	1.24	1.31	1.38	1.31	1.28
ST	59.2	59.2	66.6	66.6	66.6	52.7	58.8	65.5	53.7	55.7
PZ	33.21	33.21	33.95	33.95	33.95	36.33	37.88	38.13	38.57	36.7
Lipinski rule	Yes	Yes	Yes	Yes	Yes	Yes	Yes	Yes	Yes	Yes

MW: molecular weight (g/mol); HBA: hydrogen bond acceptor; HBD: hydrogen bond donor; LogP: lippophilicity of partition coefficient; LogS: lippopilicity of water (mg/L); PSA: polar surface area; MR: molar refractivity; Den: density (g/cm^3^ ±0.06); ST: surface tension (dyne/cm ±3.0); PZ: polarizability (cm^3^±0.5 × 10^24^); MR: molar refractivity (cm^3^ ± 0.3); ST: surface tension.

### 2.6. Molecular docking

**Figure 8.**
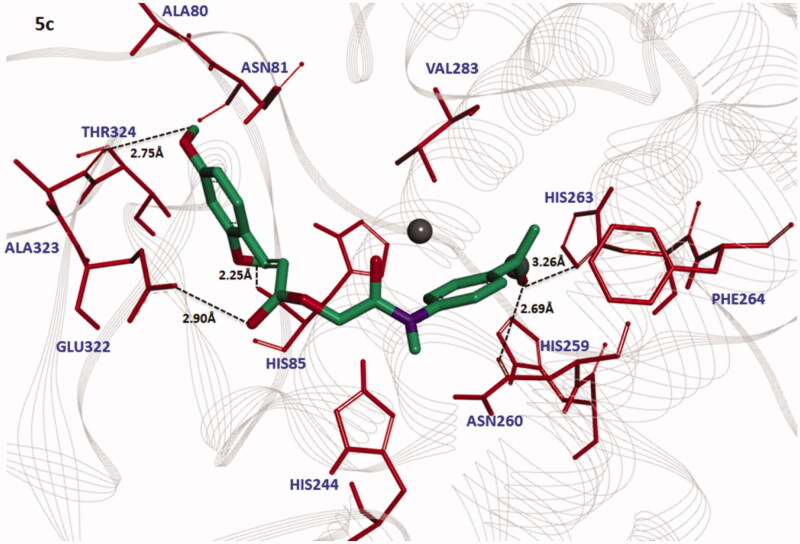
Docking interaction between **5c** and target protein. The **5c** is mentioned in green colour with embedded oxygen functional groups in red and nitrogen in purple colours. The target protein is highlighted in line ribbon format with light grey colour. The active binding site amino acids are highlighted in red colour. Two copper ions are also mentioned in black colour. Five hydrogen bonds were observed between **5c** and receptor amino acids like HIS263, ASN260, HIS85, GLU322 and ALA323 with bonding distances 3.26 Ȧ, 2.69 Ȧ, 2.25 Ȧ, 2.90 Ȧ and 2.75 Ȧ, respectively. The black dotted lines show the binding distance in angstrom (Ȧ).

The docked complexes of all the synthesized compounds (**3a**–**3e** and **5a**–**5e**) were analysed on the basis of lowest binding energy values and hydrogen bonding analyses. Results showed that **5c** was the most active compound with best binding energy value (–7.90 kcal/mol) compared to others derivatives. The graphical depiction of **5c** docking complex is mentioned in Figure 8.

The bonding analysis of all compounds against mushroom tyrosinase showed that **5c** compound is directly interacts with active residues of targeted protein. The structure activity relationship (SAR) analysis shows that **5c** builds five hydrogen bonds at specific residues (HIS263, ASN260, HIS85, GLU322 and ALA323) against the target protein. The carbonyl oxygen of benzene ring of **5c** interacts with HIS263and ASN260 positions having bonds lengths 3.26 Å and 2.69 Å. The ester oxygen of the same compound also interacts with GLU322 with bonding distance 2.90 Å. The hydroxyl groups of the same compounds form bonds with HIS85 and ALA323 with bonds length 2.25 Å and 2.75 Å, respectively. Literature study also justified that these interacted residues are significant in the downstream signalling pathways^38^. The graphical representations of all other interacted compounds (**3a**–**3e**, and **5a**, **5b**, **5d** and **5e**) against target protein are mentioned in Figures S2–S3 (see Supplementary materials).

Moreover, comparative analysis showed that **5c** has good binding energy values (−7.90 kcal/mol) as compared other derivatives. The other candidate compounds (**3a–3e**) also possess good binding energy values −7.60 kcal/mol, −7.70 kcal/mol, −7.60 kcal/mol, −7.40 kcal/mol and −7.60 kcal/mol, while **5a, 5b, 5d** and **5e** showed −7.10 kcal/mol, −6.90 kcal/mol, −6.40 kcal/mol and −7.10 kcal/mol binding energy values, respectively (Table 3). Here, it is noticeable that most of the candidate compounds bind in the active binding regions but little fluctuated with their conformational positions. The comparative binding analysis and bioassay study showed that **5c** was the most active and significant compound as compared to other synthesized compounds.

**Table 3.** Docking results of synthesized compounds using PyRx

Compounds	Energy values (kcal/mol)
**3a**	−7.6
**3b**	−7.7
**3c**	−7.6
**3d**	−7.4
**3e**	−7.6
**5a**	−7.1
**5b**	−6.9
**5c**	−7.9
**5d**	−6.4
**5e**	−7.1

## 3. Conclusion

The hydroxy substituted 2-[(4-acetylphenyl)amino]-2-oxoethyl derivatives **3a–e** and **5a–e** with various hydroxyl moieties have been synthesized to explore their role in tyrosinase inhibitory activity. The analogue **5c** showed excellent activity with IC_50_=0.0089 µM better than standard kojic acid IC_50_ =16.69 µM. The enzyme inhibitory kinetics results confirmed that compounds **5c,** a competitive inhibitor, and **5d,** a mixed type inhibitor. Both compounds **5c** and **5d** inhibit the enzyme by irreversible mode of binding. The computational molecular dynamic simulation results revealed that synthesized compounds bind well with the active binding sites and most potent derivative **5c** formed stable complex with target protein. The cytotoxicity evaluation performed against murine melanoma (B16F10) cells showed that most potent derivative **5c** is nontoxic at a dose of 12 µg/mL. The same dose of **5c** was selected to determine antimelanogenic activity; the results showed that it produced antimelenogenic effects in murine melanoma (B16F10) cells. It is proposed that analogue **5c** may serve as lead structure to design highly potent antimelanogenic agents.

## 4. Experimental

### 4.1. Synthesis of title compounds 3a–e and 5a–e

4-Actylaniline (0.01 mol) was reacted with chloroacetyl chloride (0.01 mol) in the presence of triethylamine (0.01 mol) in anhydrous dichloromethane (20 ml) at 0 to −5 °C. The reaction mixture was stirred at room temperature for 4 h, extracted with ethyl acetate, washed with dilute hydrochloric acid and 5% sodium hydroxide solution. The organic layer was removed under reduced pressure to afford the intermediate **1** as solid with melting point 154–155 °C; FTIR *ν*_max_ cm^−1^: 3178 (**–**NH), 2922 (sp^2^ C**–**H), 2812 (sp^3^ C**–**H), 1634 (C=O amide) and 1599 (C = C aromatic). The title amides **3a–e** were obtained by treating intermediate **1** with hydroxy substituted benzoic acids **2a–e** (0.01 mole), triethyl amine (0.01 mol) and potassium iodide (0.01 mol) in dimethylformamide (DMF, 10 ml) at room temperature, while compounds **5a–e** were synthesized by reacting intermediate **1** with acids **4a–e** under the same conditions. After the completion of reaction, the mixture was poured into ice cold water with stirring and extracted with ethyl acetate (4 × 20 ml). The combined organic layer was washed with dilute hydrochloric acid (5%), sodium carbonate solution (5%) and finally with aqueous NaCl solution. The organic layer was dried over anhydrous magnesium sulphate, filtered and the solvent was removed under reduced pressure to afford the crude title amides **3a–e** and **5a–e** purified by silica gel column chromatography.

#### 2-[(4-Acetylphenyl)amino]-2-oxoethyl 3-hydroxybenzoate (3a)

Solid; reaction time, 24 h; yield 76%; melting point 166–168 °C; *R*_f_ 0.52 (*n*-hexane:ethyl acetate 2:1), FTIR *ν*_max_ cm^−1^: 3336 (−OH), 3132 (−NH), 2936 (sp^2^ C–H), 2835(sp^3^ C–H), 1731 (C=O ester), 1656 (C=O amide), 1596 (C=C aromatic), 1147 (C–O, ester); ESI-MS: *m/z* 336 [M + 23] (M + Na)^+^; ^1^H NMR (400 MHz, DMSO, ppm) *δ*: 10.63 (s, 1H, −NH), 9.90 (s, 1H, −OH), 7.94 (d, *J* = 8.2 Hz, 2H, H-17, H-19), 7.74 (d, *J* = 8.2 Hz, 2H, H-16, H-20), 7.48–7.43 (m, 2H, H-1, H-6), 7.35 (t, *J* = 2.4 Hz, 1H, H-3), 7.09–7.07 (m, 1H, H-5), 4.96 (s, 2H, H-11, −CH_2_), 2.54 (s, 3H, H-23, −CH_3_); ^13 ^C NMR (100 MHz, DMSO_,_ ppm) *δ*: 196.98 (C-21, C=O, ketone), 166.47 (C-12, C=O, amide), 165.91 (C-8, C=O, ester), 158.03 (C-4), 143.28 (C-15), 132.4 (C-18), 130.8 (C-2), 130.4 (C-6), 130 (C-17,19), 121.1 (C-1), 120.6 (C-5), 120.2 (C-4), 118.9 (C-16,20), 116.3 (C-3), 60.6 (C-11, –CH_2_), 26.9 (C-23, –CH_3_); Anal Calcd For C_17_H_15_NO_5_: C, 65.17; H, 4.97; Found C, 65.14; H, 4.95.

#### 2-[(4-Acetylphenyl)amino]-2-oxoethyl 4-hydroxybenzoate (3b)

Solid; reaction time, 24 h; yield 82%; melting point 184–186 °C; *R*_f_ 0.53 (*n*-hexane:ethyl acetate 2:1), FTIR *ν*_max_ cm^−1^: 3365 (–OH), 3133 (–NH), 2983 (sp^2^ C–H), 2885(sp^3^ C–H), 1715 (C=O ester), 1648 (C=O amide), 1599 (C=C aromatic), 1152 (C–O, ester); ESI-MS: *m/z* 336 [M + 23] (M + Na)^+^; ^1^H NMR (400 MHz, DMSO, ppm) *δ*: 10.55 (s, 1H, –NH), 10.42 (s, 1H, –OH), 7.96 (d, *J* = 7.2 Hz, 2H, H-17, H-19), 7.88 (d, *J* = 4.4 Hz, 2H, H-1, H-3), 7.73 (d, *J* = 6.8 Hz, 2H, H-16, H-20), 6.89 (d, *J* = 4.4 Hz, 2H, H-5, H-6), 4.92 (s, 2H, H-11, –CH_2_), 2.54 (s, 3H, H-22, –CH_3_); ^13 ^C NMR (100 MHz, DMSO_,_ ppm); *δ* 196.98 (C-21, C=O, ketone), 166.73 (C-12, C=O, amide), 165.69 (C-5), 162.76 (C-8, C=O, ester), 143.29 (C-15), 132.43 (C-18), 132.26 (C-1,3), 130.01 (C-17,19), 120.17 (C-2), 118.98 (C-16,20), 115.89 (C-4,6), 63.27 (C-11, –CH_2_), 26.9 (C-22, –CH_3_); Anal Calcd For C_17_H_15_NO_5_: C, 65.17; H, 4.97; Found C, 65.13; H, 4.93.

#### 2-[(4-Acetylphenyl)amino]-2-oxoethyl 2,4-dihydroxybenzoate (3c)

Solid; reaction time, 24 h; yield 73%; melting point 227–229 °C; *R*_f_ 0.47 (*n*-hexane:ethyl acetate 2:1), FTIR *ν*_max_ cm^−1^: 3354 (–OH), 3155 (–NH), 2951 (sp^2^ C–H), 2856(sp^3^ C–H), 1728 (C=O ester), 1637 (C=O amide), 1597 (C=C aromatic), 1158 (C–O, ester); ESI-MS: *m/z* 352 [M + 23] (M + Na)^+^; ^1^H NMR (400 MHz, DMSO, ppm) *δ*: 10.59 (s, 1H, H-7, –OH),10.54 (s, 1H, H-8, –OH), 10.46 (s, 1H, H-15, –NH), 7.96 (d, *J* = 8.4 Hz, 2H, H-18, H-20), 7.76 (d, *J* = 8.4 Hz, 2H, H-17, H-21), 7.73 (d, *J* = 8.8 Hz, 1H, H-3), 6.43 (dd, *J_1 = _*8.8 Hz, *J_2_*=2.0 Hz, 1H, H-4), 6.34 (d, *J* = 2.0 Hz, 1H, H-6), 4.96 (s, 2H, H-12, –CH_2_), 2.54 (s, 3H, H-23, –CH_3_); ^13 ^C NMR (100 MHz, DMSO_,_ ppm) *δ*: 196.98 (C-22, C=O, ketone), 168.66 (C-13, C=O, amide), 166.36(C-9, C=O, ester), 164.95 (C-5), 163.12 (C-1), 143.18 (C-16), 132.58 (C-19), 132.51 (C-3), 130.03 (C-19,20), 119.01 (C-17,21), 108.97 (C-4), 104.33 (C-2), 103.07 (C-6), 63.37 (C-12, –CH_2_), 26.91 (C-23, –CH_3_); Anal Calcd For C_17_H_15_NO_5_: C, 62.00; H, 4.55; Found C, 61.95; H, 4.52.

#### 2-[(4-Acetylphenyl)amino]-2-oxoethyl 3,4-dihydroxybenzoate (3d)

Solid; reaction time, 24 h; yield 70%; melting point 205–207 °C; *R*_f_ 0.46 (*n*-hexane:ethyl acetate 2:1), FTIR *ν*_max_ cm^−1^: 3314 (–OH), 3156 (–NH), 2934 (sp^2^ C–H), 2843 (sp^3^ C–H), 1730 (C=O ester), 1642 (C=O amide), 1592 (C=C aromatic), 1148 (C–O, ester); ESI-MS: *m/z* 352 [M + 23] (M + Na)^+^; ^1^H NMR (400 MHz, DMSO, ppm) *δ*: 10.56 (s, 1H, H-14, –NH), 9.88(s, 1H, H-7, –OH), 9.44 (s, 1H, H-8, –OH), 7.98 (d, *J* = 5.2 Hz, 2H, H-18, H-20), 7.75 (d, *J* = 5.2 Hz, 2H, H-17, H-21), 7.43 (d, *J* = 2.0 Hz, 1H, H-2), 7.40 (dd, *J_1 = _*8.4, *J_2_*=2.0 Hz, 1H, H-6), 6.86 (d, *J* = 8.0 Hz, 1H, H-5), 4.86 (s, 2H, H-12, –CH_2_), 2.51 (s, 3H, H-23, –CH_3_); ^13 ^C NMR (100 MHz, DMSO_,_ ppm) *δ*: 196.98 (C-22, C=O, ketone), 166.78 (C-13, C=O, amide), 165.8(C-9, C=O, ester), 151.26(C-4), 145.6 (C-3), 143.32 (C-16), 132.42 (C-19), 130.01 (C-18,20), 122.69 (C-1), 120.39(C-6), 118.96 (C-17,21), 117.01 (C-5),115.85 (C-2), 63.26 (C-12, –CH_2_), 26.90 (C-23, –CH_3_); Anal Calcd For C_17_H_15_NO_5_: C, 62.00; H, 4.55; Found C, 61.96; H, 4.52.

#### 2-([(4-Acetylphenyl)amino]-2-oxoethyl 3,5-dihydroxybenzoate (3e)

solid; reaction time, 24 h; yield 74%; melting point 249–251 °C; *R*_f_ 0.45 (*n*-hexane:ethyl acetate 2:1), FTIR *ν*_max_ cm^−1^: 3365 (–OH), 3145 (–NH), 2949 (sp^2^ C–H), 2862 (sp^3^ C–H), 1744 (C=O ester), 1623 (C=O amide), 1601 (C=C aromatic), 1147 (C–O, ester); ESI-MS: *m/z* 352 [M + 23] (M + Na)^+^; ^1^H NMR (400 MHz, DMSO, ppm) *δ*: 10.57 (s, 1H, H-14, –NH), 9.69 (s, 2H, H-7, H-8 –OH), 7.97 (d, *J* = 1.6 Hz, 2H, H-18, H-20), 7.74 (d, *J* = 1.6 Hz, 2H, H-17, H-21), 6.90 (d, *J* = 2.0 Hz, 2H, H-2, H-6), 6.49 (s, 1H, H-4), 4.92 (s, 2H, H-12, –CH_2_), 2.54 (s, 3H, H-23, –CH_3_); ^13 ^C NMR (100 MHz, DMSO_,_ ppm) *δ*: 196.98 (C-22, C=O,ketone), 166.50 (C-13, C=O, amide), 165.96 (C-9, C=O, ester), 159.06 (C-3,5), 143.27 (C-16), 132.46 (C-19), 131.25 (C-18,20), 130.03 (C-1), 118.97 (C-17,21), 108.1 (C-4), 107.86 (C-2,6), 63.57 (C-12, –CH_2_), 26.90 (C-23, –CH_3_); Anal Calcd For C_17_H_15_NO_5_: C, 62.00; H, 4.55; Found C, 61.97; H, 4.51.

#### 2-[(4-Acetylphenyl)amino]-2-oxoethyl cinnamate (5a)

Solid; reaction time, 24 h; yield 78%; melting point 164–166 °C; *R*_f_ 0.52 (*n*-hexane:ethyl acetate 2:1), FTIR *ν*_max_ cm^−1^: 3144 (–NH), 2918 (sp^2^ C–H), 2820 (sp^3^ C–H), 1728 (C=O ester), 1650 (C=O amide), 1593 (C=C aromatic), 1146 (C–O, ester); ESI-MS: *m/z* 346 [M + 23] (M + Na)^+^; ^1^H NMR (400 MHz, DMSO, ppm) *δ*: 10.54 (s, 1H, H-14, –NH), 7.96 (d, *J* = 2.0 Hz, 2H, H-18, H-20), 7.75 (m, 5H, H-17, H-21, H-7, H-6, H-2), 7.46 (m, 3H, H-3, H-4, H-5), 6.78 (d, *J* = 16.0 Hz, 1H, H-8), 4.86 (s, 2H, H-12, –CH_2_), 2.54 (s, 3H, H-23, –CH_3_); ^13 ^C NMR (100 MHz, DMSO_,_ ppm) *δ*: 196.98 (C-22, C=O, ketone), 166.56 (C-13, C=O, amide), 166.27 (C-9, C=O, ester), 145.91 (C-7), 143.26 (C-16), 134.4 (C-19), 132.46 (C-1), 131.16 (C-3,5), 130.01 (C-4), 129.8 (C-2,6), 119.02 (C-17,21), 117.86 (C-8), 63.19 (C-12, –CH_2_), 26.90 (C-23, –CH_3_); Anal Calcd For C_19_H_17_NO_4_: C, 70.58; H, 5.01; Found C, 70.53; H, 4.98.

#### 2-[(4-Acetylphenyl)amino]-2-oxoethyl (E)-3-(4-hydroxyphenyl)acrylate (5b)

Solid; reaction time, 24 h; yield 72%; melting point 226–228 °C; *R*_f_ 0.45 (*n*-hexane:ethyl acetate 2:1), FTIR *ν*_max_ cm^−1^: 3366 (–OH), 3173 (–NH), 2981 (sp^2^ C–H), 2867 (sp^3^ C–H), 1732 (C=O ester), 1639 (C=O amide), 1593 (C=C aromatic), 1147 (C–O, ester); ESI-MS: *m/z* 362 [M + 23] (M + Na)^+^; ^1^H NMR (400 MHz, DMSO, ppm) *δ*: 10.57 (s, 1H, H-15, –NH), 10.11 (s, 1H, H-7, –OH), 7.95 (d, *J* = 2.0 Hz, 2H, H-19,21), 7.74 (d, *J* = 2.0 Hz, 2H, H-18, H-22), 7.63 (m, 3H, H-2, H-6, H-8), 6.83 (d, *J* = 8.4 Hz, 2H, H-3, H-5), 6.52 (d, *J* = 16.0 Hz, 1H, H-9), 4.82 (s, 2H, H-13, –CH_2_), 2.54 (s, 3H, H-25, –CH_3_); ^13^C NMR (100 MHz, DMSO_,_ ppm) *δ*: 196.99 (C-23, C=O, ketone), 166.74 (C-14, C=O, amide), 166.64 (C-10, C=O, ester), 160.56 (C-4), 146.13 (C-8), 143.32 (C-17), 132.42 (C-20), 130.96 (C-2,6), 129.99 (C-19,21), 125.46 (C-1), 118.99 (C-18,22), 116.31 (C-9), 113.87 (C-3,5), 63.0 (C-13, –CH_2_), 26.91 (C-25, –CH_3_); Anal Calcd For C_19_H_17_NO_5_: C, 67.25; H, 5.01; Found C, 67.21; H, 4.97.

#### 2-[(4-Acetylphenyl)amino]-2-oxoethyl (*E*)-3-(2,4-dihydroxyphenyl)acrylate (5c)

Solid; reaction time, 24 h; yield 70%; melting point 236–238 °C; *R*_f_ 0.43 (*n*-hexane:ethyl acetate 2:1), FTIR *ν*_max_ cm^−1^: 3366 (–OH), 3154 (–NH), 2959 (sp^2^ C–H), 2844(sp^3^ C–H), 1726 (C=O ester), 1654 (C=O amide), 1592 (C=C aromatic), 1157 (C–O, ester); ESI-MS: *m/z* 378 [M + 23] (M + Na)^+^; ^1^H NMR (400 MHz, DMSO, ppm) *δ*: 10.48 (s, 1H, H-8, –OH), 10.22 (s, 1H, H-16, –NH), 9.93 (s, 1H, H-7, –OH), 7.96 (d, *J* = 2.0 Hz, 2H, H-20, H-22), 7.85 (d, *J* = 16.0 Hz, 1H, H-9), 7.73 (d, *J* = 2.0 Hz, 2H, H-19, H-23), 7.46 (d, *J* = 8.4 Hz, 1H, H-6), 6.5 (d, *J* = 16.0 Hz, 1H, H-10), 6.38 (s, 1H, H-3), 6.3 (d, *J* = 8.4 Hz, 1H, H-5), 4.78 (s, 2H, H-14, –CH_2_), 2.54 (s, 3H, H-25, –CH_3_); ^13 ^C NMR (100 MHz, DMSO_,_ ppm) *δ*: 196.98 (C-24, C=O, ketone), 167.22 (C-15, C=O, amide), 166.91(C-11, C=O, ester), 161.69 (C-2), 159.21 (C-4), 143.32 (C-18), 141.94 (C-9), 132.41 (C-21), 131.07 (C-6), 130 (C-20,22), 118.99 (C-19,23), 116.31 (C-9), 113.01 (C-10), 112.59 (C-1), 108.38 (C-5), 102.96 (C-3), 62.86 (C-14, –CH_2_), 25.6 (C-25, –CH_3_); Anal Calcd For C_19_H_17_NO_6_: C, 64.22; H, 4.78; Found C, 64.18; H, 4.73.

#### 2-[(4-Acetylphenyl)amino]-2-oxoethyl-(*E*)-3-(4-chlorophenyl)acrylate (5d)

solid; reaction time, 24 h; yield 78%; melting point 161–163 °C; *R*_f_ 0.50 (*n*-hexane:ethyl acetate 2:1), FTIR *ν*_max_ cm^−1^: 3136 (–NH), 2923 (sp^2^ C–H), 2855 (sp^3^ C–H), 1719 (C=O ester), 1648 (C=O amide), 1591 (C=C aromatic), 1150 (C–O, ester); ESI-MS: *m/z* 382.5 [M + 23] (M + Na)^+^; ^1^H NMR (400 MHz, DMSO, ppm) *δ*: 10.52 (s, 1H, H-15, –NH), 7.96 (d, *J* = 1.6 Hz, 2H, H-19, H-21), 7.82 (d, *J* = 1.6 Hz, 2H, H-18, H-22), 7.75 (m, 3H, H-2, H-6, H-8), 7.51 (d, *J* = 6.4 Hz, 2H, H-3, H-5), 6.84 (d, *J* = 16.0 Hz, 1H, H-9), 4.85 (s, 2H, H-13, –CH_2_), 2.54 (s, 3H, H-25, –CH_3_); ^13 ^C NMR (100 MHz, DMSO_,_ ppm) *δ*: 196.97 (C-23, C=O, ketone), 166.5 (C-14, C=O, amide), 166.14 (C-10, C=O, ester), 144.48 (C-8), 143.24 (C-17), 135.69 (C-20), 133.37 (C-4), 132.47 (C-1), 130.69 (C-19,21), 130 (C-2,6), 129.48 (C-3,5), 119.01 (C-18,22), 118.69 (C-9), 60.21 (C-13, –CH_2_), 26.89 (C-25, –CH_3_); Anal Calcd For C_19_H_18_NO_4_: C, 63.42; H, 5.00; Found C, 63.38; H, 4.95.

#### 2-[(4-Acetylphenyl)amino]-2-oxoethyl 3-(4-hydroxyphenyl)propanoate (5e)

Solid; reaction time, 24 h; yield 74%; melting point 234–236 °C; *R*_f_ 0.45 (*n*-hexane:ethyl acetate 2:1), FTIR *ν*_max_ cm^−1^: 3356 (–OH), 3149 (–NH), 2944 (sp^2^ C–H), 2856(sp^3^ C–H), 1721 (C=O ester), 1643 (C=O amide), 1590 (C=C aromatic), 1147 (C–O, ester); ESI-MS: *m/z* 364 [M + 23] (M + Na)^+^; ^1^H NMR (400 MHz, DMSO, ppm) *δ*: 10.43 (s, 1H, H-15, –NH), 9.18 (s, 1H, H-7, –OH), 7.95 (d, *J* = 2.0 Hz, 2H, H-19,21), 7.71 (d, *J* = 2.0 Hz, 2H, H-18, H-22), 7.05 (d, *J* = 2.0 Hz, 2H, H-2, H-6), 6.68 (d, *J* = 2.0 Hz, 2H, H-3, H-5), 4.70 (s, 2H, H-13, –CH_2_), 2.78 (t, *J* = 7.6 Hz, 2H, H-8), 2.66 (t, *J* = 1.2 Hz, 2H, H-9), 2.54 (s, 3H, H-25, –CH_3_); ^13 ^C NMR (100 MHz, DMSO_,_ ppm) *δ*: 196.99 (C-23, C=O, ketone), 172.49 (C-10, C=O, ester), 166.52 (C-14, C=O, amide), 156.10 (C-4), 143.20 (C-17), 132.47 (C-20), 130.90 (C-2,6), 129.59 (C-19,21), 119.10 (C-18,22), 115.58 (C-3,5), 63.00 (C-13, –CH_2_), 35.66 (C-9), 29.83 (C-8), 26.90 (C-25, –CH_3_); Anal Calcd For C_19_H_19_NO_5_: C, 66.86; H, 5.57; Found C, 66.80; H, 5.52.

### 4.2. Anti-tyrosinase activity

The mushroom tyrosinase (EC 1.14.18.1) (Sigma Chemical Co., ST. Louis, MO) was used for *in vitro* bioassays as described previously with some modifications^39^^,^^40^. Briefly, 140 µL of phosphate buffer (20 mM, pH 6.8), 20 µL of mushroom tyrosinase (30 U/mL) and 20 µL of the inhibitor solution were placed in the wells of a 96-well micro plate. After pre-incubation for 10 min at room temperature, 20 µL of L-DOPA (3,4-dihydroxyphenylalanine) (0.85 mM) was added and the plate was further incubated at 25 °C for 20 min. Subsequently the absorbance of dopachrome was measured at 492 nm using a micro plate reader (OPTI _Max_, Tunable). Kojic acid was used as a reference inhibitor and for negative tyrosinase inhibitor phosphate buffer was used instead of the inhibitor solution. The extent of inhibition by the test compounds was expressed as the percentage of concentration necessary to achieve 50% inhibition (IC_50_). Each concentration was analysed in three independent experiments run in triplicate. The IC_50_ values were determined by the data analysis and graphing software Origin 8.6, 64-bit.

### 4.3. Kinetic analysis of the inhibition of mushroom tyrosinase

A series of experiments were performed to determine the inhibition kinetics by following method^41^^,^^42^. Inhibitor **5c** with concentrations 0.0, 0.0005, 0.0022, 0.0045, 0.009, 0.018 µM and **5d** with concentrations 0.0, 1.75, 3.5, 7, 14 and 28 µM, respectively, were used. Substrate L-DOPA concentration was among 0.0625–2 mM in all kinetic study. Pre-incubation and measurement time was the same as discussed in mushroom tyrosinase inhibition assay protocol. The formation of DOPAchrome was continuously monitored at 475 nm for 5 min at a 30 s interval in the microplate reader after addition of enzyme. The inhibition type on the enzyme was assayed by Lineweaver–Burk plots of inverse of velocities (1/*V*) versus inverse of substrate concentration 1/[S] mM^−1^, and the inhibition constant *Ki* was determined by the Dixon plot of 1/*V* versus inhibitor concentrations.

### 4.4. Cytotoxicity and cell morphology

To test the safety level of **5c** compound, MTT assay (3-(4,5-dimethylthiazol-2-yl)-2,5-diphenyltetrazolium Bromide) was performed^43^. The murine melanoma (B16F10) cells (1.5 × 10^5^ cells/ml) were seeded in 24 well plates (SPL Korea) with DMEM medium at 37 °C and 5% CO_2_ incubator. Test compound **5c** was dissolved in DMSO and diluted with culture medium to get the final concentrations of 0 (control), 6, 12 and 24 µg/ml, and then added to the cells for 24 h further incubation. The MTT assay was then conducted, and results were calculated in percentage of control. In addition, cellular images were taken by inverted fluorescent microscope (Nikon, ECLIPSE, Tokyo, Japan) to access the **5c** effect on cell morphology.

### 4.5. Melanin content measurement

For melanin measurement, melanoma (1.5 × 10^5^) cells were seeded in cell culture plates for 24 h at 37 °C and 5% CO_2_incubator. Then cells were incubated with various concentrations of **5c** in cell culture medium for further 24 h. Later, assay for melanin measurement was performed following already reported method^44^. Briefly, the cells were trypsinised and centrifuged for 5 min at 1000 rpm. The cell pellet was dissolved in 1 N NaOH at 60 °C for 1 h and melanin content was measured spectrophotometrically at 405 nm wavelengths using a microplate reader (BioTek, synergy HT, Winooski, VT).

### 4.6. Computational studies

#### 4.6.1. Retrieval of protein structure from PDB

The crystal structure of mushroom tyrosinase (PDBID: 2Y9X) was retrieved from the Protein Data Bank (PDB) (http://www.rcsb.org). The energy minimization of target protein was carried out by employing conjugate gradient algorithm and Amber force field in UCSF Chimera 1.10.1^45^. The stereo-chemical properties, Ramachandran graph and values^46^ of mushroom tyrosinase were assessed by Molprobity server^47^, while the hydrophobicity graph was generated by Discovery Studio 4.1 Client^48^. The protein architecture and statistical percentage values of helices, beta-sheets, coils and turns were accessed by VADAR 1.8^49^.

#### 4.6.2. Candidate structures

The synthesized compounds **3a–e** and **5a–e** were sketched in drawing ACD/ChemSketch tool. The designed ligand molecules were further visualized and minimized by UCSF Chimera 1.10.1 in PDB format. The drug assessment properties of these compounds were access by various computational tools like Molinspiration (http://www.molinspiration.com/) and Molsoft (http://www.molsoft.com/). The Osiris Property Explorer (http://www.organic-chemistry.org/prog/peo/) an online tool was used to calculate their possible tumorigenic or mutagenic risks and drug-likeness values. Lipinski’s rule of five was justified by using Molsoft and Molinspiraion tools. Furthermore, different molecular properties such as molar refractivity, density, surface tension and polarizability were also accessed by chemsketch online.

#### 4.6.3. Molecular docking

Molecular docking experiment was employed on all synthesized ligand molecules **3a–e** and **5a–e** against mushroom tyrosinase using diverse PyRx tool^50^. The grid box parametric dimension values (*X* = 61.0781, *Y* = 56.3001 and *Z* = 63.1015) with spacing 1.0Ȧ were adjusted to attain the finest binding conformational pose of protein-ligand molecules. The maximum docking poses (100 numbers of run) for each docking were adjusted to obtain the best docking complex with good conformational pose. All compounds were docked separately against crystal structure of mushroom tyrosinase and the obtained docked complexes were further evaluated on lowest binding energy (kcal/mol) values using Discovery Studio (4.1) and UCSF Chimera 1.10.1.

## Supplementary Material

Supplemental Material
