# Supplementary material for: Synthesis, computational studies, tyrosinase inhibitory kinetics and antimelanogenic activity of hydroxy substituted 2-[(4-acetylphenyl)amino]-2-oxoethyl derivatives
Source: J Enzyme Inhib Med Chem. 2019 Aug 28;34(1):1562–72. doi: 10.1080/14756366.2019.1654468 (PMC8853709; doi:10.1080/14756366.2019.1654468)
Supplement: Supplemental Material [file IENZ_A_1654468_SM7169.pdf]

Molecular dynamics simulations

Procedure

Based on docking results and biological assay study, we performed structural dynamic studies on the selected lowest energy complexes of mushroom tyrosinase. All MD simulations were carried out by Groningen Machine for Chemicals Simulations (GROMACS) 4.5.4 package [1] with GROMOS 53A6 force field and water model SPC216 to get an equilibrated structure [2]. These three best ranked energy docked complexes **5a**, **5c** and **5d** were employed for MD simulations using Gromacs 4.5.4 separately, to confirm their stability against mushroom tyrosinase. All the simulations were done in bound and unbound format. The receptor molecules were separated from docked complexes and build their topology files using GROMOS 53A6 force-field and water model SPC216. Similarly, ligand molecules topology files were generated using online automated topology builder PRODRG Server [3]. The protein-ligand complexes were then solvated with SPC216 explicit water molecules and placed in the center of a cubic box of size 24 × 24× 24 A3. We adjusted 1.0 Å distance between receptor (mushroom tyrosinase) and the edge of the simulation box to immerse the protein with water and rotate freely. Prior to minimization, the overall system charge was neutralized by adding ions. Particle Mesh Ewald (PME) was used to calculate the long-range electrostatic interactions and periodic boundary conditions were applied in all directions [4]. The steepest descent approach (1000 ps) was used for each protein-ligand complex for energy minimization (nsteps= 50000) to remove the initial steric clashes. The linear constraint solver (LINCS) [5] algorithm was used for covalent bond constraints. Further NVT [6] was performed for 100 ps to equilibrate the system with protein and ligand for constant volume, pressure (1 atm) and temperature (300 K). The final MD run was set to 5000 ps for each protein-ligand complex with nsteps 2500000, and trajectories were saved for further analysis using Xmgrace (http://plasma-gate.weizmann.ac.il/Grace/) and UCSF Chimera 1.10.1 software.

Results and discussion

To evaluate the mushroom tyrosinase flexibility and overall stability of docking complexes, we executed time dependent MD simulation at 5(ns) using Gromacs 4.5.4. The residual deviations and fluctuation in the complexes were determined by RMSD and RMSF graphs generated by using Xmgrace software. The bound and unbound simulation of **5c** was done separately to check the difference in residual backbone of target protein. Figures 1 and 2 exhibited the residual deviation and fluctuations of **5c** docked complexes respectively. An increasing trend was

observed in RMSD from 0.1 to 0.25 (nm) at time 0-1.5 ns in both bound and unbound complexes, while after that little steady fluctuation was observed throughout the simulation period. The comparative analysis showed that in all simulation period from 0-5ns little fluctuations were observed for **5c** docking complex. The predicted graph suggested that **5c** complexes for both bound and unbound is much better and little fluctuated throughout the simulation period. These predicted results justified that binding of **5c** against targeted protein is more stable compared to all other derivatives. Comparative results also justified that **5c** did not much change the conformational symmetry of target protein which confer their significance over other derivatives. The RMSF results also reflect that both C and N-terminal lobes of mushroom tyrosinase are fluctuated throughout the simulation period. The unbound graph shows higher fluctuated peaks as compared to bound condition of **5c** compound. The comparative analysis justify that **5c** is more stable and less fluctuated throughout the simulation period. Insight from MD simulations stable behaviors of **5c** docked complex throughout MD trajectories thus increasing the efficacy of docking results. All candidates' molecules showed little noticeable changes in residual fluctuation of **5a**, and **5d** at different loop regions, while is less fluctuated in case of **5c** complex.

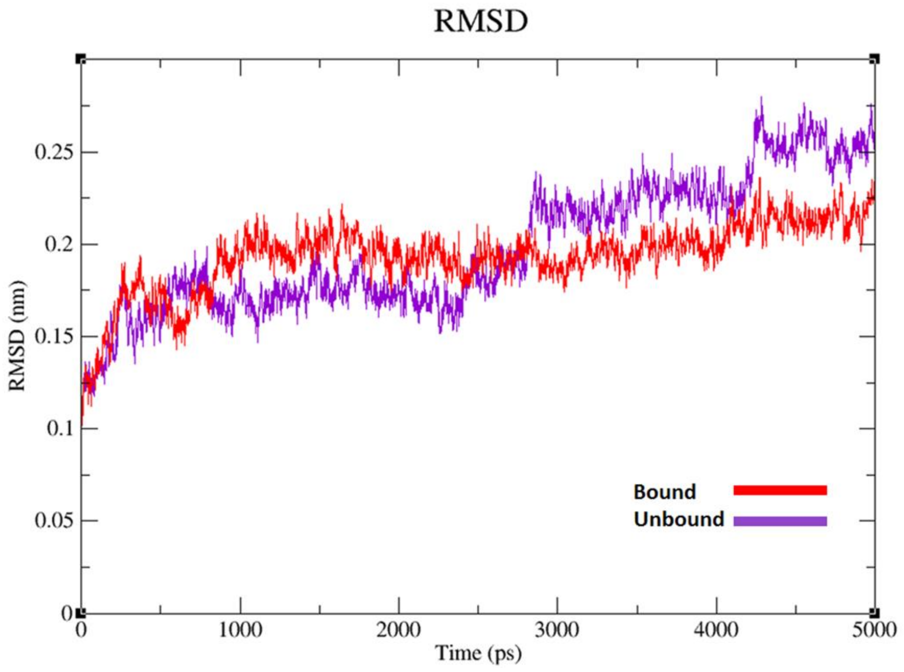

Fig. 1. RMSD graph of **5c** in bound and unbound conditions at 0-5ns time frame. The graph lines with red and purple represents the bound and unbound condition of 5c complex respectively.

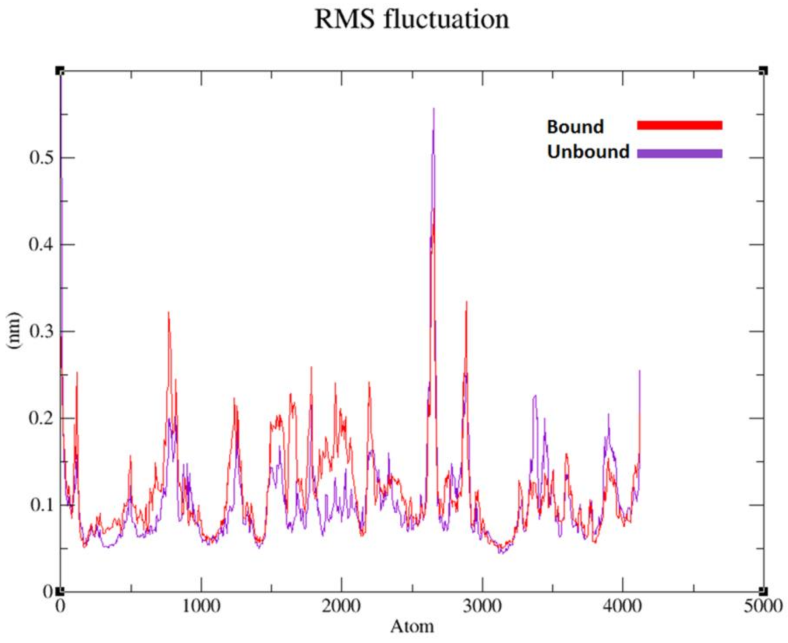

Fig. 2. RMSF graph of **5c** in bound and unbound conditions at 0-5ns time frame. The graph lines with red and purple represents bound and unbound conditions at different time (ns) of **5c** complex respectively.

The compactness of protein is measured by radius of gyration (Rg). The predicted results of both bound and unbound showed that Rg value is little fluctuated between 1.95-2.05 nm throughout the simulation time 0-5ns. These results justified residual backbone and folding of receptor protein was little steadily stable after binding the inhibitors. The Rg values and graph line of **5c** shows that protein is stabily folded and less fluctuated in docking complex (Fig.3).

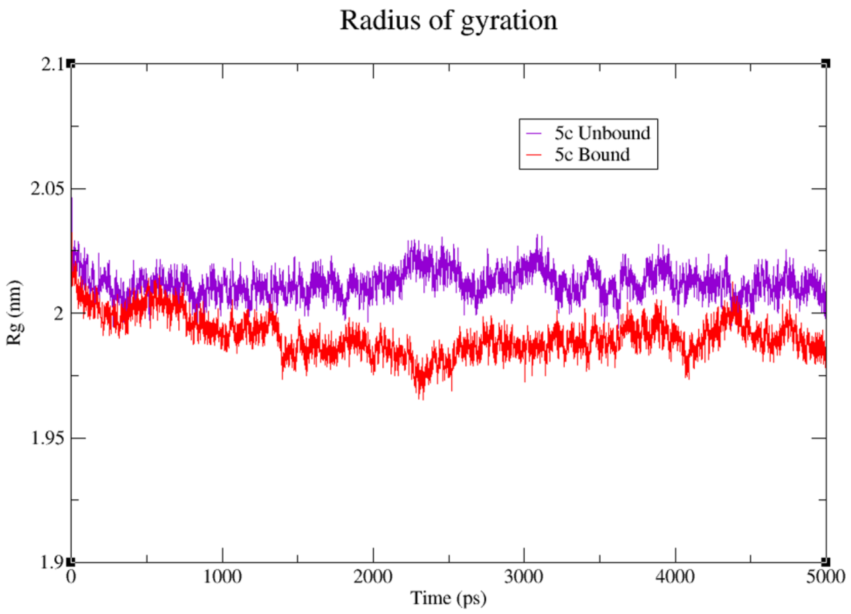

Fig. 3. Radius of gyration (Rg) graph of **5c** bound and unbound formats. The graph lines with red and purple represents the bound and unbound target protein respectively.

In computational approaches both docking and MD analysis showed that **5c** compound has more therapeutical potential against the mushroom tyrosinase as compared to other synthesized compounds.

References

1. Pronk S, Páll S, Schulz R, Larsson P, Bjelkmar P, Apostolov R, Shirts MR, Smith JC, Kasson PM, Van Der Spoel D, Hess B. GROMACS 4.5: a high-throughput and highly parallel open source molecular simulation toolkit. *Bioinformatics*. **2013**, 29(7):845-54.
2. Oostenbrink C, Villa A, Mark AE, Van Gunsteren WF. A biomolecular force field based on the free enthalpy of hydration and solvation: the GROMOS force- field parameter sets 53A5 and 53A6. *Journal of computational chemistry*. **2004**, 25(13):1656-76.
3. Schüttelkopf AW, Van Aalten DM. PRODRG: a tool for high-throughput crystallography of protein–ligand complexes. ActaCrystallographica Section D: *Biological Crystallography*. **2004,** 60(8):1355-63.
4. Wang H, Dommert F, Holm C. Optimizing working parameters of the smooth particle mesh Ewald algorithm in terms of accuracy and efficiency. *The Journal of chemical physics*. **2010**, 133(3):034117.
5. Amiri S, Sansom MS, Biggin PC. Molecular dynamics studies of AChBP with nicotine and carbamylcholine: the role of water in the binding pocket. *Protein Engineering, Design & Selection*. **2007**, 20(7):353-9.
6. Labík S, Smith WR. Scaled particle theory and the efficient calculation of the chemical potential of hard spheres in the NVT ensemble. *Molecular Simulation*. **1994**, 12(1):23-31.
